# Supplementary material for: Redistribution of EZH2 promotes malignant phenotypes by rewiring developmental programmes
Source: EMBO Rep. 2019 Aug 29;20(10):e48155. doi: 10.15252/embr.201948155 (PMC6776892; doi:10.15252/embr.201948155)
Supplement: Supplementary file 1 — Appendix [file EMBR-20-e48155-s001.pdf]

APPENDIX

Appendix Figure S1. Characterization of genetically modified cell lines.. 2

Appendix Table S1. Cell culture conditions. Growth media and source of all cell lines used in this study 3

Appendix Table S2. Oligonucleotides. Sequence and description of all oligonucleotides used in this study… 4

Appendix Table S3. Non-glioma NCI60 cancer cell lines. Names of cell lines that correspond to the numbers shown in Fig S2F. 5

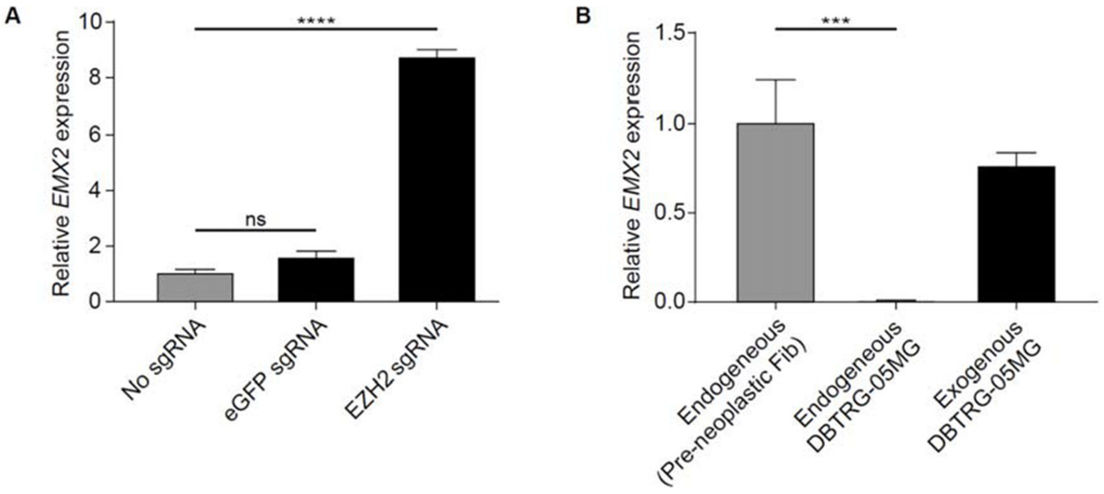

Appendix Figure S1: Characterization of genetically modified cell lines

A. Quantification by qRT-PCR of *EMX2* relative levels in transformed cells after CRISPR-Cas9 mediated knock-out of EZH2 or an eGFP control. Values represent mean ± SEM from three technical replicates. Four asterisks indicate p-value < 0.0001 (one-way ANOVA followed by Dunnet's multiple comparisons test).

B. Quantification by qRT-PCR of relative *EMX2* levels in DBTRG-05MG GBM cells, untransduced (endogenous) or transduced with pTRIPZ-EMX2 (exogenous). Pre-neoplastic fibroblasts were used as a standard for endogenous *EMX2* levels in non-transformed cells. Note that expression of the doxycycline-inducible pTRIPZ construct was not induced and the detected mRNA represents leaky expression. To avoid possible aspecific effects due to protein overexpression, uninduced cells expressing endogenous-like levels of *EMX2* were used for *in vivo* assays. Values represent mean ± SEM from three technical replicates. Three asterisks indicate p-value < 0.001 (one-tailed unpaired Student's t-test).

Appendix Table S1. Cell culture conditions. Growth media and source of all cell lines used in this study.

Cell Line	Medium	FBS	L-glutamine (mM)	Pen/Strep (mM)	Other Supplements	Source
Untransformed immortalised fibroblasts	MEM	15%	100	100	-	Scaffidi and Misteli (2012)
Pre-neoplastic immortalised fibroblasts	MEM	15%	100	100	-	Scaffidi and Misteli (2012)
Transformed immortalised fibroblasts	MEM	15%	100	100	-	Scaffidi and Misteli (2012)
DBTRG-05MG	RPMI 1640	10%	100	100	25mM HEPES	Crick Institute Repository
M059K	DMEM	10%	100	100	-	Crick Institute Repository
U-87 MG	DMEM	10%	100	100	1% non-essential AA	Crick Institute Repository
T98G	MEM	10%	100	100	-	Crick Institute Repository
AGS	Ham’s F12	10%	-	100	-	Crick Institute Repository
Patient-derived xenografts (LXFL1674, LXFA677, SXF1301, MAXFMX1, MEXF535, GXA3067, PAXF1990)	RPMI 1640	10%	100	100	25mM HEPES	Crick Institute Repository
HEK293T	DMEM	10%	100	100	-	Crick Institute Repository
H1299	RPMI 1640	10%	100	100	25mM HEPES	Crick Institute Repository
LN-229	DMEM	10%	100	100	-	Crick Institute Repository
HCT116	DMEM	10%	100	100	-	Crick Institute Repository
A549	DMEM	10%	100	100	-	Crick Institute Repository
Phoenix	DMEM	10%	100	100	-	Crick Institute Repository

MEM: Minimum Essential Medium
RPMI: Roswell Park Memorial Institute media
DMEM: Dulbecco's Modified Eagle's Medium
FBS: Fetabl Bovine Serum
Pen/Strep: Penicillin/Streptomycin

Appendix Table S2. Oligonucleotides. Sequence and description of all oligonucleotides used in this study.

Oligonucleotide	Sequence (5'-3')
EMX2 quantification RT-qPCR/ChIP-PCR Forward	CACACCCCCTATTCGCCTC
EMX2 quantification RT-qPCR/ ChIP-PCR Reverse	CCAGATATCGGTAGCGGTGG
Cyclophillin quantification RT-qPCR Forward	GTCAACCCCACCGTGTTCTT
Cyclophillin quantification RT-qPCR Reverse	CTGCTGTCTTTGGGACCTTGT
HOXB9 quantification RT-qPCR Forward	GGAAACTTCGGCGGGCA
HOXB9 quantification RT-qPCR Reverse	TTTTTCCGGGAAGAGCGAGC
HOXB9 ChIP-PCR RT-qPCR Forward	CTGTCCCAGACCACTTGTCC
HOXB9 ChIP-PCR RT-qPCR Reverse	GCCAGGAGAGTCCGAATGAG
EZH2 CRISPR KO test Forward	ACAGGAAACGATTGCCATCC
EZH2 CRISPR KO test Reverse	GCACAAATGAGCACCTTTCTG
GAPDH ChIP-PCR RT-qPCR Forward	TCCAATTCCCCATCTCAGTC
GAPDH ChIP-PCR RT-qPCR Reverse	GCAGCAGGACACTAGGGAGT
LYPD4 Methylation test primer Forward	GATATAAGGAAAGGGGTGTAATTTGAGG
LYPD4 Methylation test primer Reverse	CCTTCCCTTCTACCAACTTCTTAAC
TNFRSF21 Methylation test primer Forward	TTGAGGAGAATTAGGGAGGAGGATT
TNFRSF21 Methylation test primer Reverse	AAAACACCCTCTCCCTCCCC
SPO11 Methylation test primer Forward	TTAAATTATTTTAAGGGATTTTAGGTTTAA
SPO11 Methylation test primer Reverse	TCCTCAAAACAACCAACAAAAACTC
PPP4R4 Methylation test primer Forward	GGTTATATGGAGGATTTGTAGGAGTT
PPP4R4 Methylation test primer Reverse	CCCTTAAAAATAAAAATCCAAACTAAAA
CALB2 Methylation test primer Forward	TTGTTATTGTTAGTAATAATAAGTGGTGGA
CALB2 Methylation test primer Reverse	CAAAATACTTCCATATTTCCAAAAACTAAA
POU6F2 Methylation test primer Forward	AGAAGTTTAGATTTTAGGGGAAGTTG
POU6F2 Methylation test primer Reverse	AACACTAAACAAAAAACCAAATAAC
EZH2/H3K27me3 WT1.1 ChIP RT-qPCR Forward	GGGATGAGAAACCAACCTGA
EZH2/H3K27me3 WT1.1 ChIP RT-qPCR Reverse	GGGTTAGAACAAGGACAGGG
EZH2/H3K27me3 WT1.2 ChIP RT-qPCR Forward	TTTAGATGGGTTGCCGAGTT
EZH2/H3K27me3 WT1.2 ChIP RT-qPCR Reverse	GGGTCCGGCTTGAAGTTATA

Appendix Table S3: Non-glioma NCI60 cancer cell lines. Names of cell lines that correspond to the numbers shown in Fig S2F.

**Cell line number**	**NCI60 cell line name**
1	MALME.3M
2	RPMI.8226
3	M14
4	UO.31
5	NCI.H23
6	HOP.62
7	OVCAR.3
8	UACC.257
9	OVCAR.4
10	SK.MEL.2
11	BT.549
12	NCI.H522
13	T.47D
14	K.562
15	SN12C
16	NCI.H460
17	A549.ATCC
18	SK.OV.3
19	HS.578T
20	SK.MEL.28
21	PC.3
22	IGROV1
23	EKVX
24	UACC.62
25	CAKI.1
26	ACHN
27	RXF.393
28	SK.MEL.5
29	NCI.H226
30	786.0
31	HL.60.TB
32	SW.620
33	DU.145
34	HOP.92
35	HCC.2998
36	MCF7
37	MDA.MB.231
38	MOLT.4
39	OVCAR.5
40	NCI.H322M
41	MDA.MB.435
42	CCRF.CEM
43	OVCAR.8
44	HT29
45	A498
46	MDA.N
47	COLO.205
48	LOX.IMVI
49	NCI.ADR.RES
50	HCT.15
51	TK.10
52	SR
53	HCT.116
54	KM12
