# Supplementary material for: Redistribution of EZH2 promotes malignant phenotypes by rewiring developmental programmes
Source: EMBO Rep. 2019 Aug 29;20(10):e48155. doi: 10.15252/embr.201948155 (PMC6776892; doi:10.15252/embr.201948155)
Supplement: Supplementary file 2 — Expanded View Figures PDF [file EMBR-20-e48155-s002.pdf]

Expanded View Figures

Figure EV1. Characterisation of EZH2 redistribution during neoplastic transformation.

A Correlation heatmap of EZH2 ChIP-seq replicates from untransformed (UT), pre-neoplastic (PN) and transformed (TR) cells. Correlation values are generated by comparing EZH2 read density over a set of EZH2 peaks common to all samples.

B Percentage of EZH2 binding sites in each cellular state that have > 25% overlap with a H3K27me3 enriched region. UT, untransformed; PN, pre-neoplastic; TR, transformed.

C ChIP-seq signal at a representative locus showing consistent EZH2 and H3K27me3 profiles. ChIP-seq signal normalised to sequencing depth is shown. Tracks are scaled to be of the same height to make samples comparable. Blue bars represent regions called as an EZH2 binding site. UT, untransformed; PN, pre-neoplastic; TR, transformed.

D Volcano plot showing the relative enrichment of EZH2 binding at all sites detected in either untransformed and pre-neoplastic (left) or pre-neoplastic and transformed cellular states (right). Numbers within the volcano plot indicate differential binding sites with a *P*-value ≤ 1e-20 and a fold change in normalised tag count ≥ 1.5. Enrichment significance calculated based on a Poisson distribution using the “getDifferentialPeaks” function in homer (see Materials and Methods). *P*-values of > 1e-100 were set to 1e-100 for display reasons.

E Quantification of EZH2 binding sites detected in a third biological replicate of EZH2 ChIP-seq. Common: EZH2 binding sites detected in both untransformed and transformed cells, UT differential: large-magnitude EZH2 binding sites enriched in untransformed cells (see Fig 1D), TR differential: large-magnitude EZH2 binding sites enriched in transformed cells (see Fig 1D).

F Quantification of EZH2 binding sites overlapping CpG islands. Common: EZH2 binding sites detected in both untransformed and transformed cells, UT differential: large-magnitude EZH2 binding sites enriched in untransformed cells (see Fig 1D), TR differential: large-magnitude EZH2 binding sites enriched in transformed cells (see: Fig 1D). EZH2 ChIP-seq signal (right) showing a representative example of a CpG island where an EZH2 binding site is lost on transformation. ChIP-seq signal normalised to sequencing depth is shown. UT, untransformed; TR, transformed. The significance of the depletion of differential sites compared to the common ones is shown (two-tailed Fisher’s exact test).

G Quantification of EZH2 binding sites overlapping lamin-associated domains (LADs). Common: EZH2 binding sites detected in both untransformed and transformed cells, UT differential: large-magnitude EZH2 binding sites enriched in untransformed cells (see Fig 1D), TR differential: large-magnitude EZH2 binding sites enriched in transformed cells (see Fig 1D). Lamin-associated domain location in human fibroblasts sourced from (Guelen *et al* [45]). The significance of the depletion or enrichment of differential sites compared to the common ones is shown (two-tailed Fisher’s exact test).

© 2019 The Authors

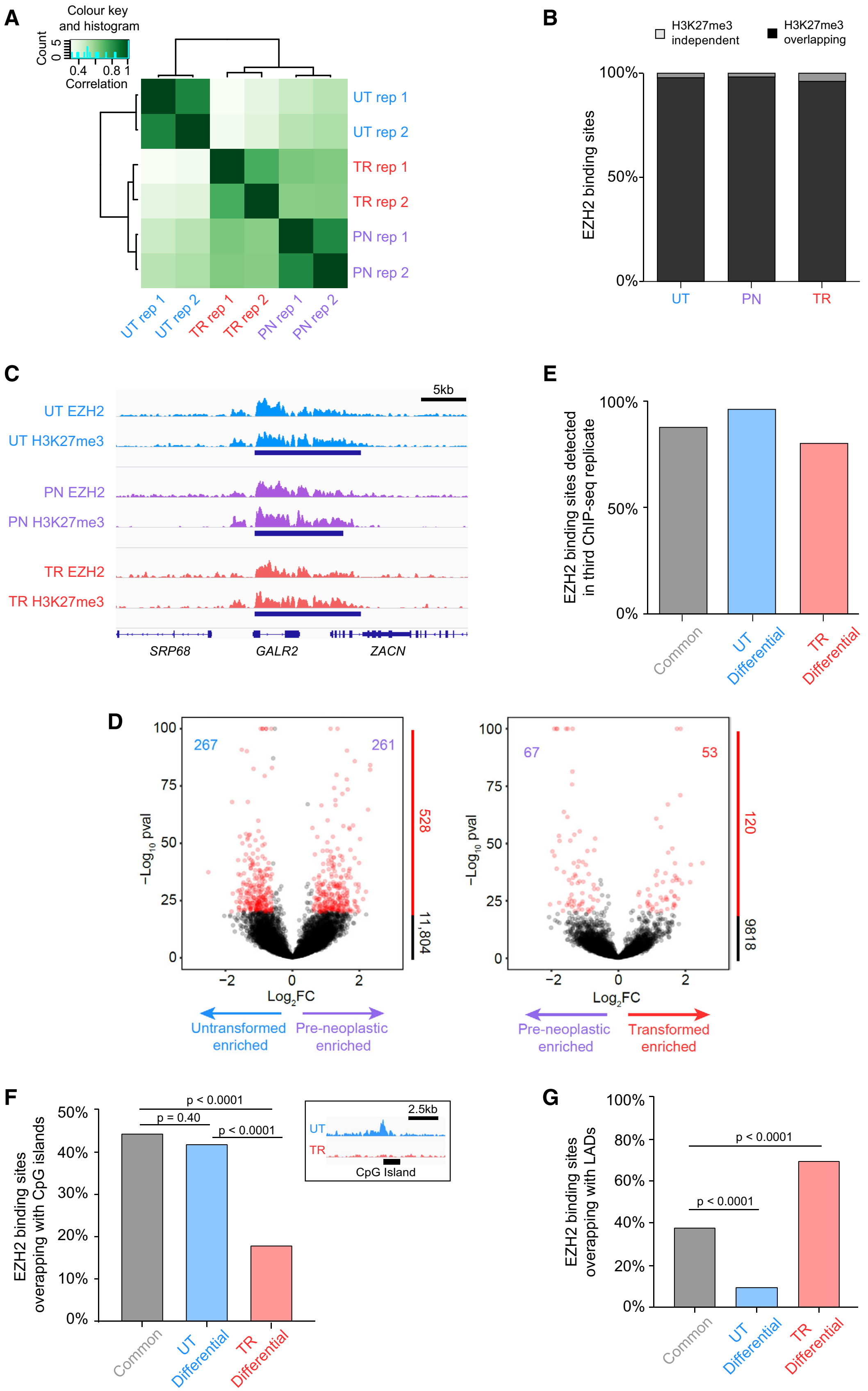

Figure EV1.

 © 2019 The Authors

Figure EV2. High DNA methylation levels at many EZH2 target genes.

A Principle component analysis of RNA-seq data from the indicated untreated and EZH2i-treated cells. UT, untransformed; PN, pre-neoplastic; TR, transformed.

B Number of EZH2-bound genes which show differential expression (FDR ≤ 0.01, $Log_2FC \geq 1$ and maxTPM ≥ 1) upon EZH2i treatment (sensitive) or remain unaltered (insensitive). UT, untransformed; PN, pre-neoplastic; TR, transformed.

C Average density profile of EZH2 and H3K27me3 at the indicated genes. Light red and grey areas represent the average profile's SEM.

D Bisulphite sequencing analysis comparing DNA methylation of promoter-associated CpG islands at representative EZH2-bound genes sensitive or insensitive to EZH2i. Each row represents an individual sequenced DNA molecule. Black and white circles represent methylated and unmethylated CpGs, respectively. For display reasons, only the first 30 CpGs of CALB2 are shown, and the remaining CpGs are also unmethylated.

E Average DNA methylation at the indicated sets of genes in the indicated NCI60 glioma cancer cell lines. Data sourced from the National Cancer Institute NCI60 cancer cell line dataset. In the boxplot, the top, middle and bottom box delimiters represent the 75th, 50th and 25th percentiles of the data, respectively. Top and bottom whiskers show the 75th percentile + 1.5*interquartile range and 25th percentile – 1.5*interquartile range, respectively. N: 256 and 843 for EZH2i sensitive and insensitive genes, respectively. Four asterisks indicate P-value < 0.0001 (two-tailed unpaired Student's t-test corrected for multiple comparison using Holm's method).

F Average DNA methylation at the indicated sets of genes in NCI60 non-glioma cancer cell lines. Cell lines from breast, lung, colon, leukaemia, melanoma, ovarian and prostate cancer are shown. In the boxplot, the top, middle and bottom box delimiters represent the 75th, 50th and 25th percentiles of the data, respectively. Whiskers are omitted for clarity. N: 256 and 843 for EZH2i sensitive and insensitive genes, respectively. The identity of the cell lines is indicated in Appendix Table S3. All cell lines have a P-value < 0.05 (two-tailed unpaired Student's t-test corrected for multiple comparison using Holm's method) with the exception of cell lines labelled with "*n*".

G *CDKN2A*/p16 expression in the indicated cellular states, untreated or treated with EZH2i (left). The expression values represent mean ± SEM from three biological replicates. ChIP-seq signal for EZH2 and H3K27me3 across the *CDKN2A* locus (right). ChIP-seq signal normalised to sequencing depth is shown. Tracks are scaled to be of the same height to make samples comparable. UT, untransformed; PN, pre-neoplastic; TR, transformed.

© 2019 The Authors

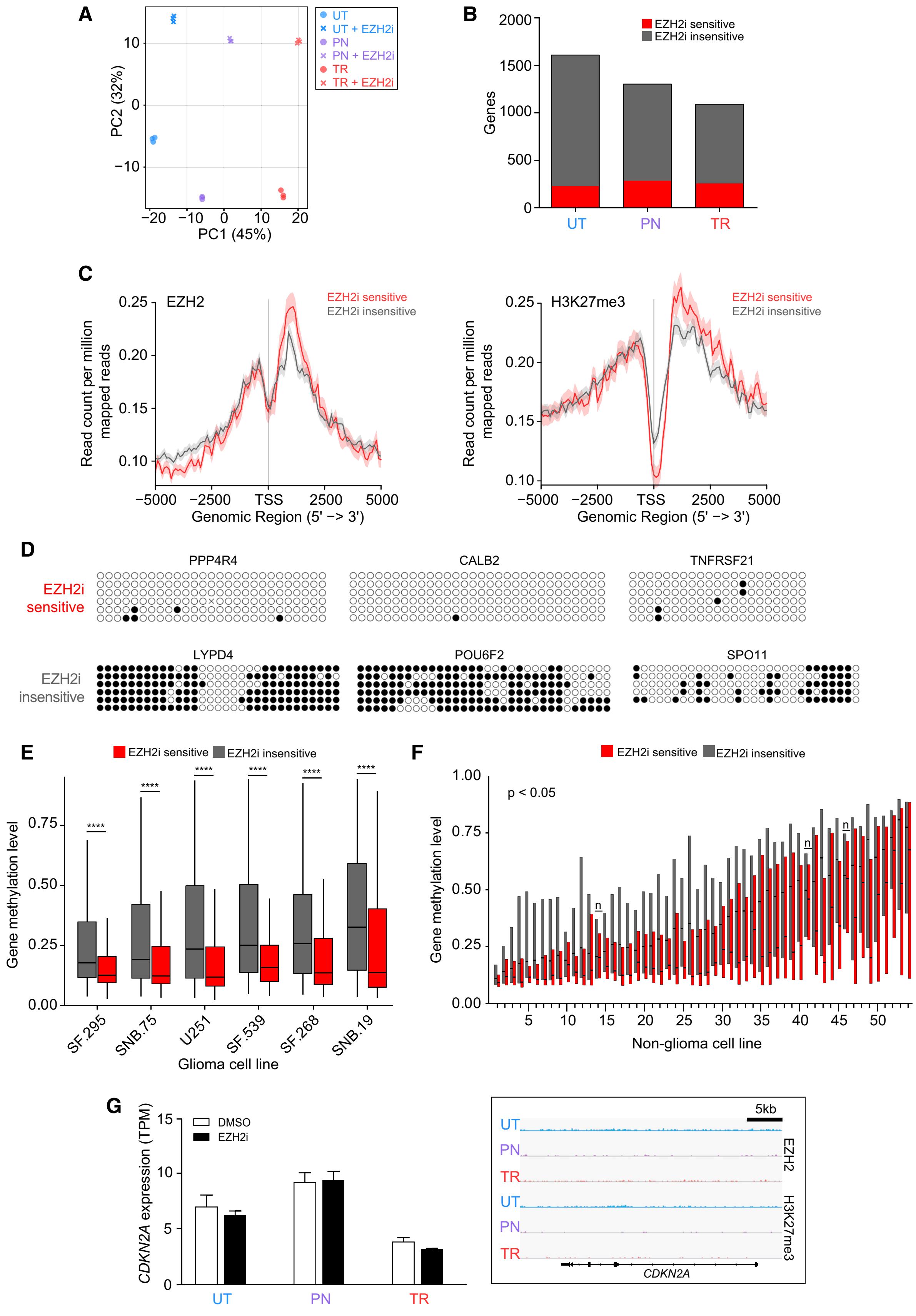

Figure EV2.

 © 2019 The Authors

Figure EV3. General de-repression of *HOX* genes in glioma.

A EZH2 ChIP-seq signal at *HOX* loci in the indicated cellular states. ChIP-seq signal normalised to sequencing depth is shown. Tracks are scaled to be of the same height to make samples comparable. Only protein coding *HOX* genes are labelled. Note the signal reduction in transformed cells especially at posterior *HOX* genes (HOXA9-A13, HOXB8-B13, HOXC11-C10). UT, untransformed; PN, pre-neoplastic; TR, transformed.

B The expression levels of all *HOX* genes in glioma cell lines as detected by RNA-seq. Data sourced from the CCLE. Numbers above bars represent cell lines in which the expression of the corresponding genes is detected (Reads per kilobase of transcript per million (RPKM) ≥ 1). Data were not available for *HOXD8*. In the boxplot, the top, middle and bottom box delimiters represent the 75th, 50th and 25th percentiles of the data, respectively. Top and bottom whiskers show the 75th percentile + 1.5*interquartile range and 25th percentile – 1.5*interquartile range, respectively. *N*: 62 glioma cell lines.

C The expression levels of *HOXA10*, *HOXB7*, *HOXC10* and *HOXD10*, chosen as representative genes of the different HOX clusters, in tumour or adjacent normal regions laser microdissected from human GBM tumours as detected by RNA-seq. Similar patterns were observed for nine other HOX genes. Data sourced from the Ivy Glioblastoma Atlas. The significance of the differential expression in normal and tumour regions is indicated (two-way ANOVA). Bars represent mean ± SEM. *N*: 3 regions sampled for each GBM tumour, 2, 3, 2, 3, 2, 3, 3 and 1 regions for normal tissue of patients 1–8, respectively. FPKM, fragments per kilobase of transcript per million.

D The expression of HOX genes in glioma patient samples, grouped by tumour grade, as detected by RNA-seq. The expression data were not available for *HOXA3, HOXA9, HOXB1, HOXC6*, *HOXC12* and *HOXD12*. Data sourced from the Chinese Glioma Genome Atlas. In the boxplot, the top, middle and bottom box delimiters represent the 75th, 50th and 25th percentiles of the data, respectively. Top and bottom whiskers show the 75th percentile + 1.5*interquartile range and 25th percentile – 1.5*interquartile range, respectively. One asterisk indicates *P*-value < 0.05 (Kruskal–Wallis test corrected for multiple comparison using Holm's method). *N*: 109 - grade II, 72 - grade III, 144 - grade IV. RPKM, reads per kilobase of transcript per million.

© 2019 The Authors

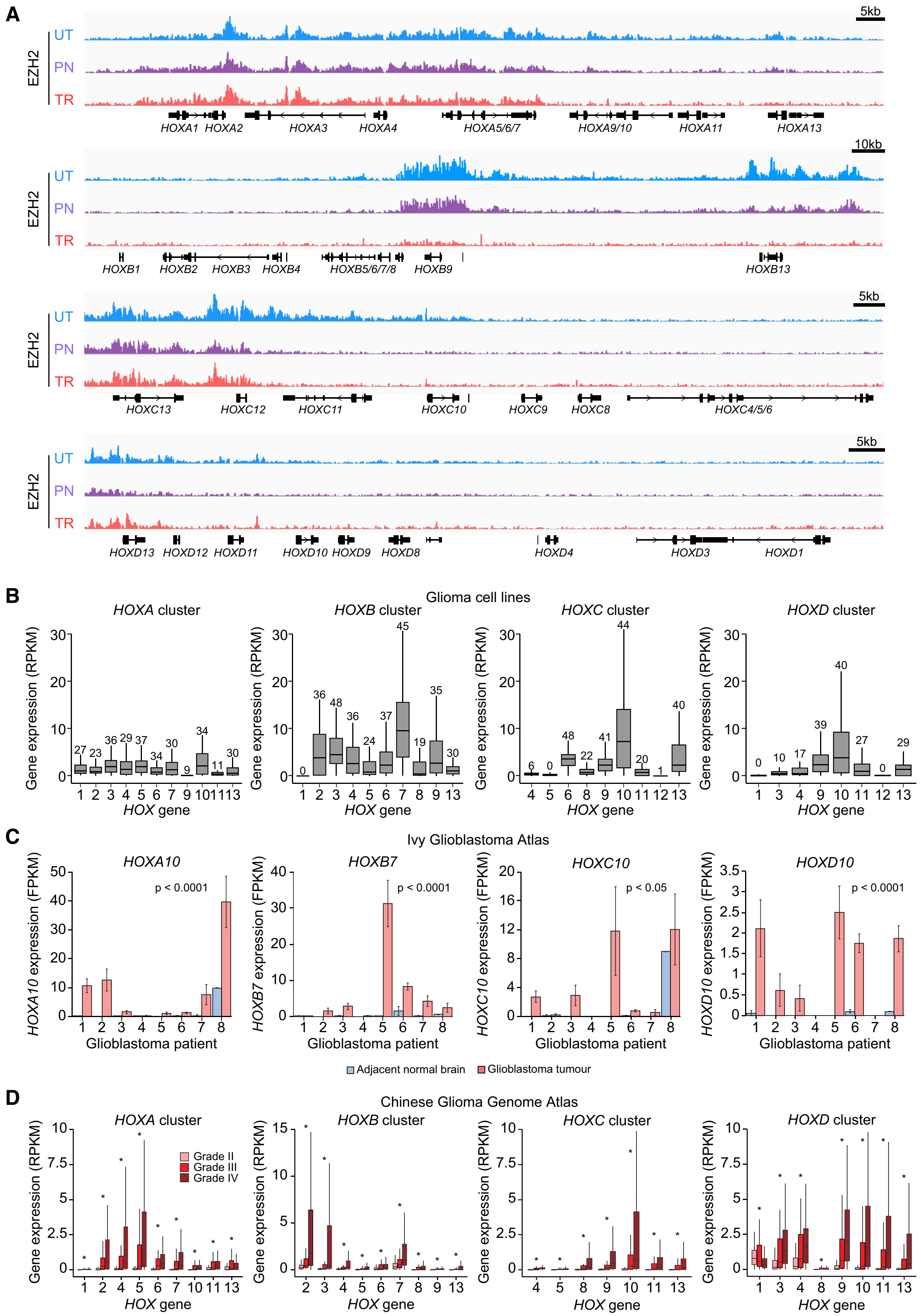

Figure EV3.

© 2019 The Authors

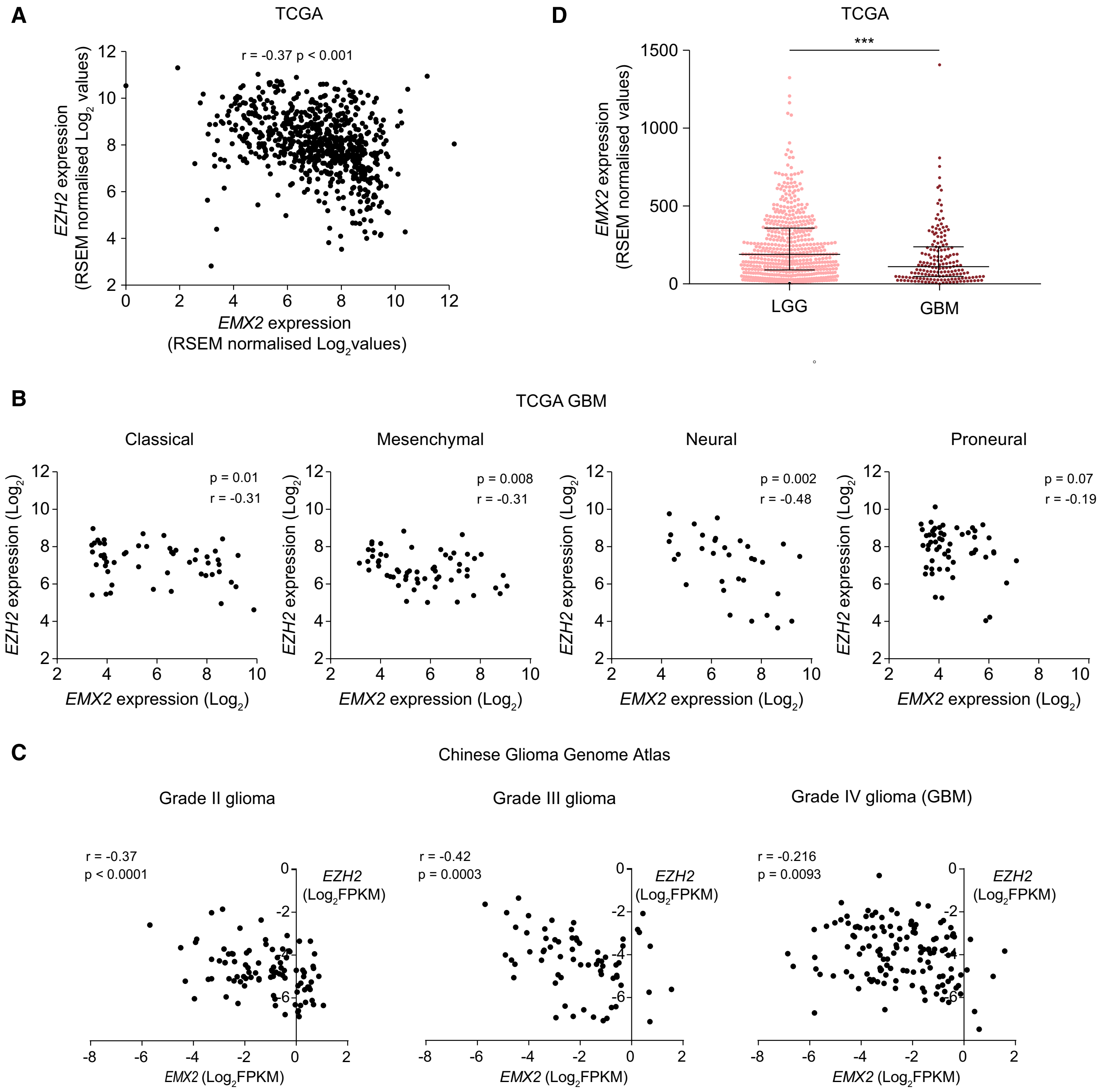

Figure EV4. Anti-correlation between *EZH2* and *EMX2* levels in glioma.

A Covariance between *EMX2* and *EZH2* expression levels in glioma patient samples [GBM and low-grade glioma (LGG)], as detected by RNA-seq. Data sourced from The Cancer Genome Atlas (TCGA). *P*-value and correlation coefficient (*r*) of the covariance are shown (Spearman rank correlation). Every dot is a patient. *N*: 702. RSEM, RNA-Seq by Expectation Maximisation.

B Covariance between *EMX2* and *EZH2* expression levels in the indicated molecular subtypes of GBM defined by Verhaak *et al* [36], as detected by microarray analysis. Data sourced from TCGA. *P*-value and correlation coefficient (*r*) of the covariance are shown (Spearman rank correlation). Every dot is a patient. *N*: 53 for classical, 58 for mesenchymal, 33 for neural, 57 for proneural.

C Covariance between *EMX2* and *EZH2* expression levels in glioma patient samples as detected by RNA-seq. Data sourced from the Chinese Glioma Genome Atlas. *P*-value and correlation coefficient (*r*) of the covariance are shown (Spearman rank correlation). Every dot is a patient. *N*: 109 for grade II, 72 for grade III, 144 for grade IV. Samples with *EMX2* or *EZH2* expression of 0 are not displayed in the Log_2 transformed graphs, but are included in significance calculations.

D The expression of *EMX2* in LGG (left) and GBM (right) patient samples, as detected by RNA-seq. Data sourced from TCGA. Three asterisks indicate *P*-value < 0.001 (one-tailed unpaired Student's *t*-test). Bars represent median ± interquartile range. *N*: 530 for LGG, 172 for GBM. RSEM, RNA-Seq by Expectation Maximisation.

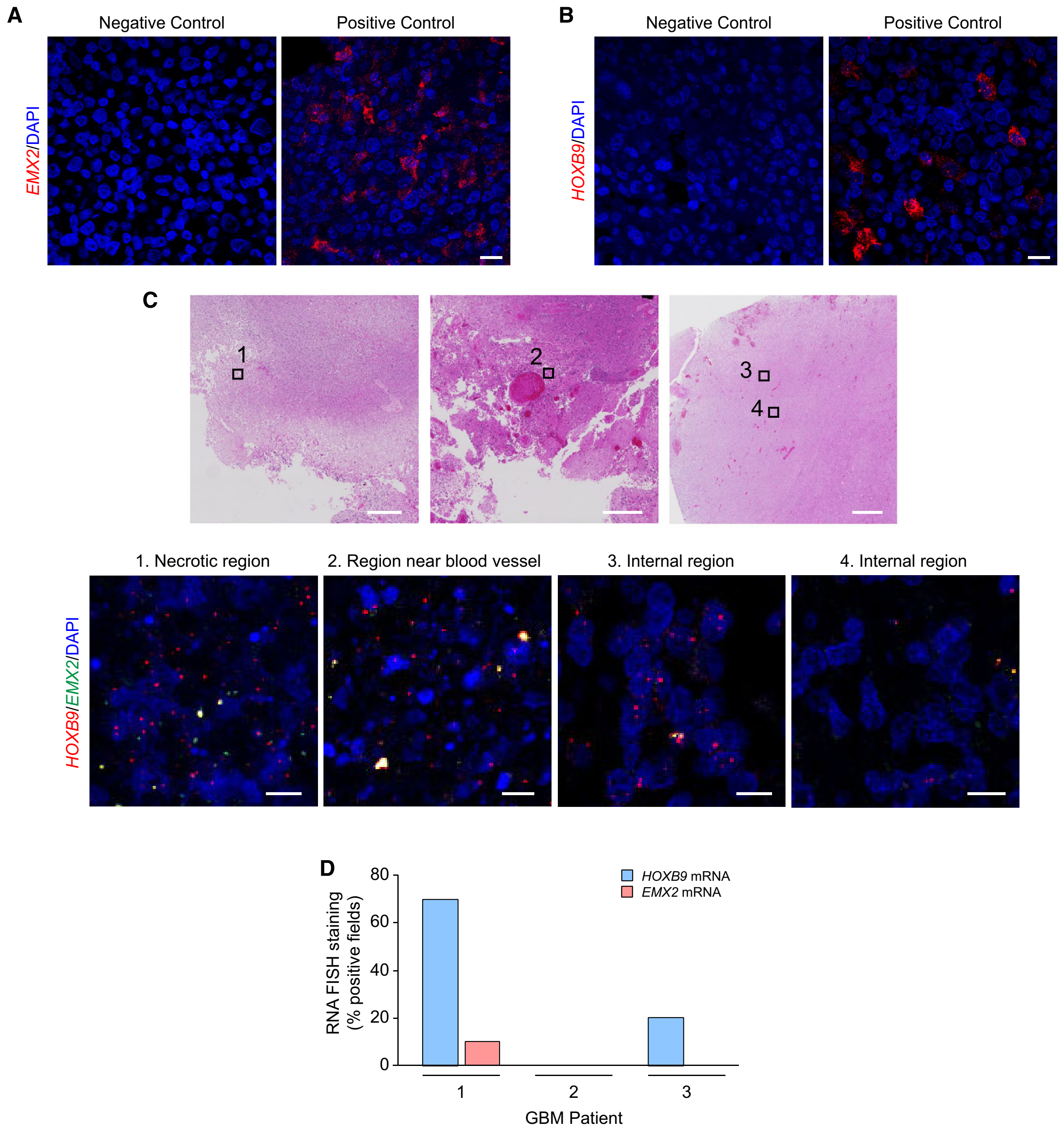

Figure EV5. *In situ* detection of *EMX2* and *HOXB9* mRNA in GBM patient samples.

A, B Visualisation of *EMX2* (left) and *HOXB9* (right) mRNA by RNA FISH. Cell pellets expressing or not expressing the genes were used as positive and negative controls for mRNA detection. Composition of cell pellets is indicated in the Materials and Methods section. Nuclei were counterstained with DAPI. Scale bar: 20 µm.

C Visualisation of *HOXB9* (red) and *EMX2* (green) mRNA by RNA FISH in various regions of GBM tumours. Serial sections were stained with haematoxylin and eosin (H&E) (top) and probed by RNA FISH (bottom). Black squares in H&E images indicate the approximate location of the fields shown below. Note the absence of correlation between *HOXB9* expression and histological features. *EMX2* levels are low or undetectable in all regions. Nuclei were counterstained with DAPI. Scale bars: 10 µm (fluorescence), 500 µm (H&E).

D Quantification of *HOXB9* and *EMX2* mRNA staining intensity in human GBM tumours. Variable percentages of *HOXB9*+ fields show intra- and inter-patient heterogeneity in expression. Ten images were acquired for each tumour sample.

 © 2019 The Authors
